# Metformin and esophageal cancer risk in Taiwanese patients with type 2 diabetes mellitus

**DOI:** 10.18632/oncotarget.13390

**Published:** 2016-11-16

**Authors:** Chin-Hsiao Tseng

**Affiliations:** ^1^ Department of Internal Medicine, National Taiwan University College of Medicine, Taipei, Taiwan; ^2^ Division of Endocrinology and Metabolism, Department of Internal Medicine, National Taiwan University Hospital, Taipei, Taiwan; ^3^ Division of Environmental Health and Occupational Medicine of the National Health Research Institutes, Zhunan, Taiwan

**Keywords:** esophageal cancer, diabetes mellitus, metformin, Taiwan

## Abstract

This study evaluated whether metformin might reduce esophageal cancer risk. Patients with type 2 diabetes mellitus diagnosed during 1999–2005 were recruited from the reimbursement database of Taiwan's National Health Insurance. Those newly treated with metformin (*n* = 288013, “ever users of metformin”) or other antidiabetic drugs (*n* = 16216, “never users of metformin”) were followed until December 31, 2011. Sensitivity analyses were conducted in a matched-pair sample of 16216 never users and 16216 ever users. Hazard ratios were estimated by Cox regression incorporated with the inverse probability of treatment weighting using propensity score. The risk associated with infection of *Helicobacter pylori*, Epstein-Barr virus, hepatitis B virus and hepatitis C virus was also evaluated. Results showed that the incidence of esophageal cancer in ever and never users was 25.03 and 50.87 per 100,000 person-years, respectively. The overall hazard ratio (95% confidence intervals) of 0.487 (0.347–0.684) suggested a significantly lower risk among metformin users. Hazard ratios comparing the first (< 21.47 months), second (21.47–46.00 months) and third (> 46.00 months) tertile of cumulative duration of metformin use to never users was 1.184 (0.834–1.680), 0.403 (0.276–0.588) and 0.113 (0.071–0.179), respectively. Infection of *Helicobacter pylori* (but not the other viral infections) significantly increased the risk, which could be ameliorated by metformin. Analyses in the matched sample consistently supported a protective role of metformin. In conclusion, metformin reduces esophageal cancer risk when the cumulative duration is more than approximately 2 years.

## INTRODUCTION

There are two main types of esophageal cancer, i.e., squamous cell carcinoma and adenocarcinoma [1]. Esophageal squamous cell carcinoma has a poor prognosis and the highest incidence occurs in Eastern Asia and Eastern and Southern Africa [1]. The risk factors of esophageal squamous cell carcinoma are not well characterized. While poor nutrition, low intake of fruits and vegetables and drinking hot beverages have been identified in Asian countries [1, 2], alcohol and smoking account for 90% of the cases in Western countries [1]. In Taiwan, the incidence of esophageal cancer is increasing steadily [3] and squamous cell carcinoma represents 91% of all cases in men and 76% in women [4]. Esophageal adenocarcinoma, more common in Western countries, is associated with obesity and gastroesophageal reflux disease [1, 2].

In patients with type 2 diabetes mellitus (T2DM), metformin reduces the risk of several types of cancer, including thyroid cancer [5], oral cancer [6], colon cancer [7], kidney cancer [8], bladder cancer [9], prostate cancer [10], breast cancer [11], endometrial cancer [12], ovarian cancer [13] and cervical cancer [14]. However, whether metformin may reduce the risk of esophageal cancer remains to be answered. Two previous studies concluded a null association. In the 1:10 case-control study using the UK-based General Practice Research Database, metformin did not significantly alter the risk in either the analysis of all patients (3819 cases and 38190 controls) or in patients with diabetes (370 cases and 3700 controls) [15]. While compared to those with no prior use, the adjusted odds ratio (95% confidence interval) for patients who had received metformin prescription of 1–14, 15–29 and ≥ 30 times was 0.99 (0.74–1.33), 1.01 (0.71–1.43) and 1.23 (0.92–1.65), respectively, in the analysis of all patients; and was 0.95 (0.68–1.33), 1.02 (0.69–1.50) and 1.31 (0.93–1.85), respectively, in the analysis of diabetes patients [15]. Another retrospective cohort study using the reimbursement database of the Taiwan's National Health Insurance (NHI) estimated an adjusted hazard ratio of 0.44 (95% confidence interval: 0.07–2.61) [16].

By using the Taiwan's NHI database, the present study further explored whether metformin use in patients with T2DM might reduce the risk of esophageal cancer. The tertile cutoffs of cumulative duration were used to evaluate a dose-response relationship, with the consideration of some infections, i.e., *Helicobacter pylori* (HP), hepatitis B virus (HBV), hepatitis C virus (HCV) and Epstein-Barr virus (EBV) [17–20]. Only patients with newly diagnosed diabetes and incident users of metformin were recruited to reduce the “prevalent user bias” [21]. To reduce “immortal time bias” when the outcome can not occur during the initial period of follow-up [21, 22], patients should have been prescribed antidiabetic drugs for at least two times, and those who were followed up for < 180 days were excluded. To reduce the confounding from the differences in baseline characteristics, Cox regression models incorporated with the inverse probability of treatment weighting (IPTW) using propensity score (PS) were created [23, 24] and sensitivity analyses were conducted in a matched-pair sample.

## RESULTS

**Figure 1:**
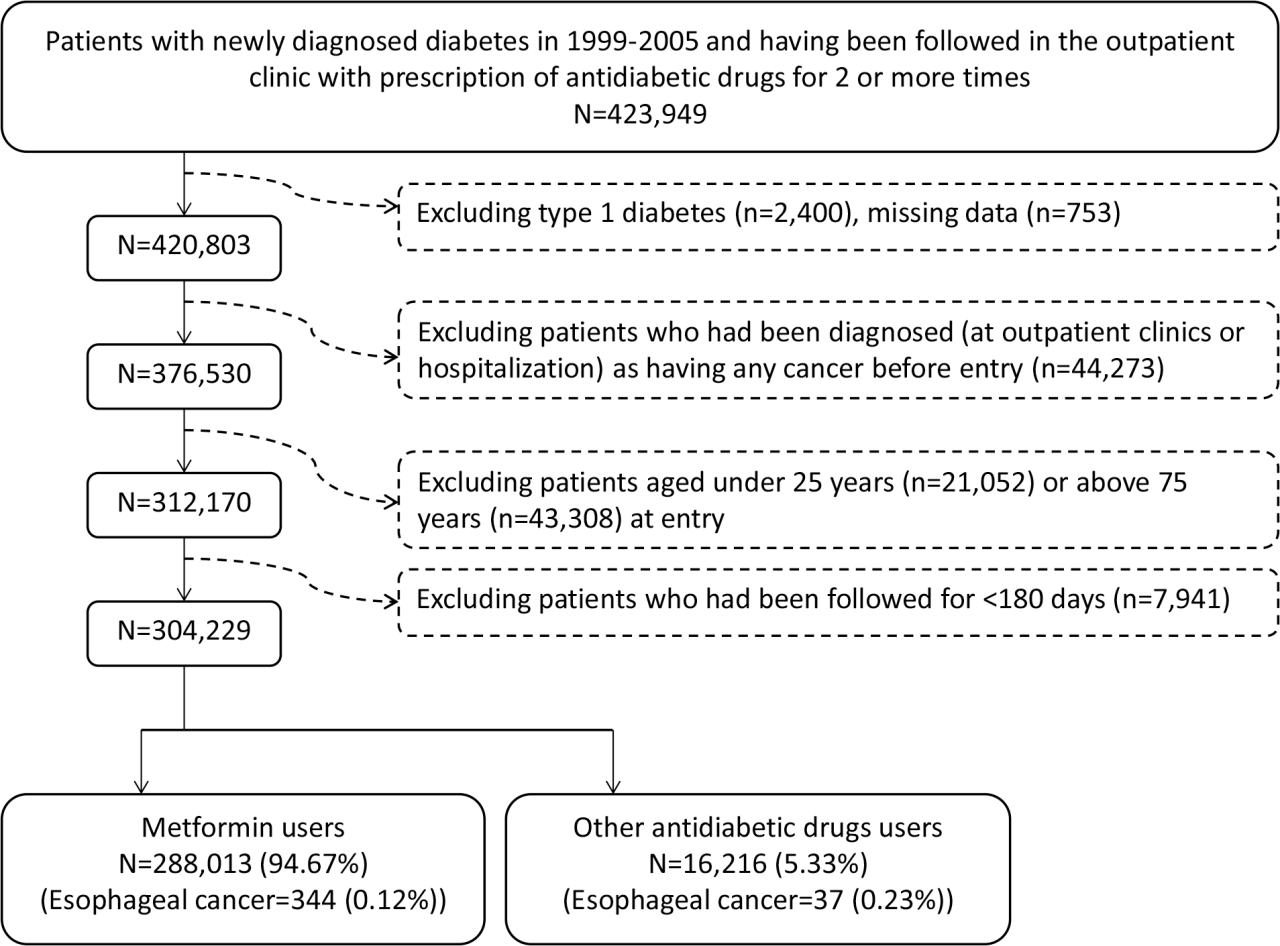
Flowchart showing the procedure in selecting the original sample into the study

There were 16216 never users and 288013 ever users of metformin in the original sample (Figure 1). All baseline characteristics differed significantly, except for peripheral arterial disease, pioglitazone and EBV-related diagnoses (Table 1). Ever users were characterized by younger age, less males, higher proportions of dyslipidemia, obesity, eye disease, and tobacco abuse, lower proportions of hypertension, nephropathy, stroke, ischemic heart disease, chronic obstructive pulmonary disease, alcohol-related diagnoses, HP infection, HBV infection and HCV infection, higher proportions of use of rosiglitazone, statin, fibrate and non-steroidal anti-inflammatory drugs (NSAID), but lower proportions of using other antidiabetic medications, angiotensin converting enzyme inhibitor/angiotensin receptor blocker (ACEI/ARB) and aspirin. The baseline characteristics were more comparable in the matched sample and only 6 variables (i.e., age, dyslipidemia, obesity, eye disease, insulin, and sulfonylurea) differed significantly. While examining the standardized differences, 14 of the 31 variables had values > 10% in the original sample, but none in the matched sample. Therefore, the results derived from the matched sample would be less influenced by residual confounding from the baseline characteristics.

**Table 1:** Baseline characteristics between metformin never users and ever users in the original sample and the propensity score matched sample

Variable	Original sample						Matched sample					
	Never users (*n* = 16216)		Ever users (*n* = 288013)		*P* value	SD	Never users (*n* = 16216)		Ever users (*n* = 16216)		*P* value	SD
	*n*	%	*n*	%			*n*	%	*n*	%		
**Demographic data**												
Age (years)*	59.14 ± 10.39		56.62 ± 10.24		< 0.0001	−25.27	59.14 ± 10.39		59.38 ± 9.73		0.0346	3.09
Sex (men)	9296	57.33	155140	53.87	< 0.0001	−7.21	9296	57.33	9466	58.37	0.0559	1.95
Occupation												
I	6329	39.03	116167	40.33	< 0.0001		6329	39.03	6370	39.28	0.5632	
II	3234	19.94	65964	22.90		7.51	3234	19.94	3133	19.32		−1.66
III	3405	21.00	56175	19.50		−3.73	3405	21.00	3424	21.11		0.51
IV	3248	20.03	49707	17.26		−7.50	3248	20.03	3289	20.28		0.60
**Living region**												
Taipei	5452	33.62	97282	33.78	< 0.0001		5452	33.62	5407	33.34	0.4247	
Northern	1656	10.21	34437	11.96		5.73	1656	10.21	1671	10.30		0.25
Central	2840	17.51	51313	17.82		0.78	2840	17.51	2852	17.59		0.16
Southern	2807	17.31	46184	16.04		−3.43	2807	17.31	2710	16.71		−1.37
Kao-Ping and Eastern	3461	21.34	58797	20.41		−2.22	3461	21.34	3576	22.05		1.90
**Major comorbidities**												
Hypertension	11971	73.82	198361	68.87	< 0.0001	−11.52	11971	73.82	11972	73.83	0.9899	0.41
Dyslipidemia	9821	60.56	197361	68.53	< 0.0001	17.35	9821	60.56	9636	59.42	0.0360	−1.90
Obesity	361	2.23	13022	4.52	< 0.0001	12.78	361	2.23	308	1.90	0.0384	−2.47
**Diabetes-related complications**												
Nephropathy	4130	25.47	46186	16.04	< 0.0001	−25.40	4130	25.47	4076	25.14	0.4904	−1.44
Eye disease	1524	9.40	41631	14.45	< 0.0001	15.68	1524	9.40	1401	8.64	0.0171	−3.08
Stroke	4017	24.77	54802	19.03	< 0.0001	−14.86	4017	24.77	4089	25.22	0.3558	1.09
Ischemic heart disease	6203	38.25	97962	34.01	< 0.0001	−9.42	6203	38.25	6224	38.38	0.8104	0.45
Peripheral arterial disease	2512	15.49	45880	15.93	0.1370	1.10	2512	15.49	2618	16.14	0.1067	1.65
**Antidiabetic drugs**												
Insulin	1351	8.33	6096	2.12	< 0.0001	−29.68	1351	8.33	1008	6.22	< 0.0001	−9.60
Sulfonylurea	11786	72.68	189783	65.89	< 0.0001	−11.24	11786	72.68	12290	75.79	< 0.0001	8.16
Meglitinide	1339	8.26	10346	3.59	< 0.0001	−21.18	1339	8.26	1257	7.75	0.0934	−1.69
Acarbose	1831	11.29	14526	5.04	< 0.0001	−22.55	1831	11.29	1850	11.41	0.7394	−0.60
Rosiglitazone	480	2.96	12955	4.50	< 0.0001	8.50	480	2.96	475	2.93	0.8695	−0.48
Pioglitazone	401	2.47	7015	2.44	0.7650	0.36	401	2.47	421	2.60	0.4798	0.35
**Potential risk factors of cancer**												
COPD	6504	40.11	110748	38.45	< 0.0001	−3.89	6504	40.11	6439	39.71	0.4611	−0.67
Tobacco abuse	262	1.62	5916	2.05	0.0001	3.37	262	1.62	268	1.65	0.7927	0.31
Alcohol-related diagnoses	1036	6.39	15452	5.37	< 0.0001	−4.88	1036	6.39	1026	6.33	0.8200	−0.55
History of HP infection	3650	22.51	54092	18.78	< 0.0001	−10.16	3650	22.51	3637	22.43	0.8627	−0.23
EBV-related diagnoses	95	0.59	1756	0.61	0.7039	0.27	95	0.59	88	0.54	0.6038	−0.45
HBV infection	339	2.09	4681	1.63	< 0.0001	−3.90	339	2.09	334	2.06	0.8456	−0.24
HCV infection	720	4.44	9396	3.26	< 0.0001	−6.61	720	4.44	701	4.32	0.6062	−0.59
**Medications that are commonly used in diabetes patients and may affect cancer risk**												
ACEI/ARB	9591	59.15	163631	56.81	< 0.0001	−5.24	9591	59.15	9584	59.10	0.9370	0.01
Calcium channel blocker	9024	55.65	141412	49.10	< 0.0001	−13.83	9024	55.65	9115	56.21	0.3088	1.23
Statin	6419	39.58	127142	44.14	< 0.0001	9.48	6419	39.58	6313	38.93	0.2281	−1.17
Fibrate	4427	27.30	92699	32.19	< 0.0001	10.97	4427	27.30	4287	26.44	0.0795	−1.74
Aspirin	7645	47.14	133306	46.28	0.0326	−2.12	7645	47.14	7572	46.69	0.4166	−0.69
NSAID	16133	99.49	287004	99.65	0.0008	2.71	16133	99.49	16129	99.46	0.7584	−0.08

* Age is expressed as mean ± standard deviation

SD: standardized difference, COPD: chronic obstructive pulmonary disease, HP: *Helicobacter pylori*, EBV: Epstein-Barr virus, HBV: hepatitis B virus, HCV: hepatitis C virus, ACEI/ARB: angiotensin converting enzyme inhibitor/angiotensin receptor blocker, NSAID: non-steroidal anti-inflammatory drugs (excluding aspirin)

Table 2 shows the incidences and hazard ratios by metformin exposure. The respective number of incident esophageal cancer in ever users and never users in the original sample was 344 and 37, with respective incidence of 25.03 and 50.87 per 100,000 person-years. There was a trend of decreasing incidence with longer cumulative duration. The overall hazard ratios showed a significantly lower risk associated with metformin in either the original sample or the matched sample. When analyzed by the tertiles of cumulative duration, a reduced risk was observed mainly for the second and third tertiles, or after a cumulative duration of approximately 2 years.

**Table 2:** Incidences of esophageal cancer and hazard ratios by metformin exposure

Metformin use	n	N	Person-years	Incidence rate(per 100,000 person-years)	HR	95% CI	*P* value
**I. Original sample**							
Never users	37	16216	72733.53	50.87	1.000		
Ever users	344	288013	1374345.55	25.03	0.487	(0.347–0.684)	< 0.0001
Tertiles of cumulative duration of metformin therapy (months)							
Never users	37	16216	72733.53	50.87	1.000		
< 21.47	210	95183	344813.60	60.90	1.184	(0.834–1.680)	0.3455
21.47–46.00	99	94864	472599.13	20.95	0.403	(0.276–0.588)	< 0.0001
> 46.00	35	97966	556932.83	6.28	0.113	(0.071–0.179)	< 0.0001
**II. Matched sample**							
Never users	37	16216	72733.53	50.87	1.000		
Ever users	22	16216	77211.50	28.49	0.557	(0.329–0.944)	0.0298
Tertiles of cumulative duration of metformin therapy (months)							
Never users	37	16216	72733.53	50.87	1.000		
< 21.47	15	5355	19409.77	77.28	1.490	(0.816–2.720)	0.1945
21.47–45.93	6	5344	26465.42	22.67	0.439	(0.185–1.040)	0.0614
> 45.93	1	5517	31336.31	3.19	0.063	(0.009–0.460)	0.0064

HR: hazard ratio, CI: confidence interval

Table 3 shows the separate effects of some infections (i.e., HP, EBV, HBV and HCV in Model I) and the joint effect of metformin and HP infection (Model II). In Model I, only HP infection was associated with a significantly higher risk. Metformin seemed to further reduce the risk in either the patients with or without HP infection (Model II).

**Table 3:** The effects of some infections and the joint effect of metformin and Helicobacter pylori infection on the risk of esophageal cancer in patients with type 2 diabetes mellitus

Model	n	N	Person-years	Incidence rate(per 100,000 person-years)	HR	95% CI	*P* value
**Model I. Separate effect of infection**							
History of HP infection							
No	268	246487	1187121.28	22.58	1.000		
Yes	113	57742	259957.80	43.47	1.493	(1.188–1.876)	0.0006
History of EBV-related diagnoses							
No	378	302378	1438471.85	26.28	1.000		
Yes	3	1851	8607.23	34.85	1.280	(0.410–3.996)	0.6703
History of HBV infection							
No	372	299209	1426369.10	26.08	1.000		
Yes	9	5020	20709.98	43.46	1.192	(0.612–2.321)	0.6063
History of HCV infection							
No	358	294113	1402059.96	25.53	1.000		
Yes	23	10116	45019.12	51.09	1.373	(0.893–2.111)	0.1486
**Model II. Joint effect of metformin and HP infection**							
Metformin (−)/HP infection (+)	12	3650	14896.45	80.56	1.000		
Metformin (+)/HP infection (+)	101	54092	245061.34	41.21	0.583	(0.319–1.064)	0.0789
Metformin (−)/HP infection (−)	25	12566	57837.07	43.22	0.694	(0.347–1.388)	0.3023
Metformin (+)/HP infection (−)	243	233921	1129284.21	21.52	0.389	(0.216–0.700)	0.0017

All models were created from the original sample with adjustment for all covariates in Table 1. Follow-up started on January 1, 2006 and ended on December 31, 2011.

HR: hazard ratio, CI: confidence interval

HP: *Helicobacter pylori*, EBV: Epstein-Barr virus, HBV: hepatitis B virus, HCV: hepatitis C virus

## DISCUSSION

The findings strongly suggested that metformin significantly reduced the risk of esophageal cancer (Tables 2 and 3). Such a protective effect was consistently observed in the original sample and the matched sample, and in a dose-response pattern (Table 2). Furthermore, HP infection was associated with an increased risk, which could be attenuated by metformin (Table 3).

The mechanisms by which metformin reduces the risk of esophageal cancer remains to be explored. In general, metformin may exert its anticancer effect through the inhibition of tumor angiogenesis [25], suppressing cancer cell metabolism [26], activation of apoptosis and autophagy [27], inhibition of mammalian target of rapamycin (mTOR) [28], immunomodulation by increasing the number of CD8^+^ tumor-infiltrating lymphocytes [29], and impairing one-carbon metabolism acting like an antifolate drug [30]. Some *in vitro* and *in vivo* studies supported these potential mechanisms. Metformin may inhibit esophageal cancer cell proliferation, both through an activation of 5′-adenosine monophosphate-activated protein kinase followed by the inhibition of mTOR/p70S6K/pS6 signaling, and through upregulation of USP7, a positive regulator of tumor suppressor p53 [31, 32]. Additionally, metformin blocks cell cycle in G0/G1 phase in esophageal cancer cell lines by reducing the expression of cyclin D1, Cdk4 and Cdk6 [33], and causes autophagy and apoptosis by downregulating Stat3 (signal transducer and activator of transcription 3), resulting in a reduced expression of Bcl-2 [34].

Although the UK study evaluated a dose-response relationship by using three categories of prescriptions, i.e., 1–14, 15–29 and ≥ 30, it suffered from the inherent limitations of a case-control design [15]. The previous Taiwanese study used a retrospective cohort design, but it suffered from limitations of small numbers of esophageal cancer (6 and 21 incident cases in comparator group and metformin users, respectively) and lack of sufficient power for a dose-response analysis [16]. Furthermore, both studies did not adjust for HP infection, and did not consider the effects of “prevalent user bias” and “immortal time bias” [21, 22].

The present study has several strengths. First, the diagnoses were considered from all sources of claims records including outpatient visits and hospital admission. Second, most medical co-payments can be waived by the NHI in patients with cancer, and there is a low drug cost-sharing in patients with certain conditions (e.g. low-income), veterans or prescription refills for chronic disease. Therefore, the detection rate of esophageal cancer would be less biased by social classes. Third, self-reporting bias could be reduced by the use of medical records.

Some limitations should be mentioned here. First, infection with human papillomavirus is a possible risk factor of esophageal cancer [17, 18]. However, this infection was not considered in the analysis because only 8 patients with such a diagnosis could be identified from the NHI database. Second, although none of the standardized differences had a value > 10%, the use of insulin and sulfonylurea remained statistically significant in the matched sample (Table 1). Both have been previously linked to a significantly higher risk of cancer in our patients with T2DM [35] and might potentially exert a confounding. However, their impacts should be minimal because of the following reasons: analysis after excluding insulin users did not change the results (data not shown), and a higher proportion of sulfonylurea use in ever users of metformin would only underestimate the protective effect of metformin if sulfonylurea did increase the risk of esophageal cancer. Other limitations included a lack of actual measurement data for confounders such as anthropometric factors, smoking, alcohol drinking, family history, lifestyle, nutritional status, dietary pattern, history of drinking hot beverages and genetic parameters. In addition, we could not evaluate the impact of biochemical data. Another limitation is the lack of information on the pathology, grading and staging of esophageal cancer. Because squamous cell carcinoma represents 91% and 76% of all cases of esophageal cancer in men and women, respectively, in Taiwan [4], the findings should better be applied to squamous cell carcinoma rather than to adenocarcinoma.

In summary, this is the first study that clearly shows a risk reduction of esophageal cancer associated with metformin use, especially after 2 years of its use. Furthermore, HP infection is an important risk factor and metformin may attenuate such a risk association.

## MATERIALS AND METHODS

The NHI implemented in Taiwan since March 1995 is a compulsory and universal system of health insurance. It covers > 99% of Taiwan residents and has contracts with > 98% of the hospitals nationwide. The reimbursement databases are handled by the National Health Research Institutes and can be used for academic researches after proposal review and approval by an ethic review board. This study was granted with an approval number 99274.

Individuals were de-identified for the protection of privacy. Diabetes was coded 250.XX and esophageal cancer 150, based on the *International Classification of Diseases, Ninth Revision, Clinical Modification* (ICD-9-CM).

Figure 1 shows the procedures in selecting a cohort of patients with newly diagnosed T2DM into the study (original sample). The patients should have been diagnosed as having diabetes at an onset age of 25–74 years during the period from 1999 to 2005. Patients with diabetes mellitus diagnosed during 1996–1998 were excluded to assure a first diagnosis of diabetes after 1999, and they should have been followed up in the outpatient clinic with prescription of antidiabetic drugs for 2 or more times (*n* = 423949). In Taiwan, patients with type 1 diabetes can be waived of much of the co-payment after a certified diagnosis with issuance of a so-called “Severe Morbidity Card”. These patients with type 1 diabetes (*n* = 2400) were excluded because metformin is not indicated for them. Patients with missing data (*n* = 753), with a diagnosis of any cancer before entry (*n* = 44273), aged < 25 (*n* = 21052) or ≥ 75 (*n* = 43308) years, and followed up for < 180 days (*n* = 7941) were also excluded.

Cumulative duration (months) of metformin use was calculated and its tertiles were used for analyses. Demographic data of age, sex, occupation and living region, and factors that might be correlated with metformin use, diabetes severity or cancer risk were considered as potential confounders. The living region and occupation were classified as detailed elsewhere [7]. In brief, the living region was classified as Taipei, Northern, Central, Southern, and Kao-Ping/Eastern. Occupation was classified as class I (civil servants, teachers, employees of governmental or private businesses, professionals and technicians), class II (people without a specific employer, self-employed people or seamen), class III (farmers or fishermen) and class IV (low-income families supported by social welfare, or veterans).

Other confounders included 1) major comorbidities associated with diabetes mellitus: hypertension (ICD-9-CM code: 401–405), dyslipidemia (272.0–272.4) and obesity (278); 2) diabetes-related complications: nephropathy (580–589), eye disease (250.5, 362.0, 369, 366.41 and 365.44), stroke (430–438), ischemic heart disease (410–414), and peripheral arterial disease (250.7, 785.4, 443.81 and 440–448); 3) antidiabetic drugs: insulin, sulfonylurea, meglitinide, acarbose, rosiglitazone and pioglitazone; 4) potential risk factors of cancer: chronic obstructive pulmonary disease (a surrogate for smoking; 490–496), tobacco abuse (305.1, 649.0 and 989.84), alcohol-related diagnoses (291, 303, 535.3, 571.0–571.3 and 980.0), history of HP infection (defined below), diagnoses related to EBV infection (075, 710.3 and 710.4), HBV infection (070.22, 070.23, 070.32, 070.33 and V02.61) and HCV infection (070.41, 070.44, 070.51, 070.54 and V02.62); and 5) medications that are commonly used in diabetes patients and may potentially affect cancer risk: ACEI/ARB, calcium channel blocker, statin, fibrate, aspirin and NSAID (excluding aspirin). History of HP infection was defined based on one of the following two criteria: 1) having received an HP eradication therapy (detailed previously [36] and defined in brief as a combination use of proton pump inhibitors or H2 receptor blockers, plus clarithromycin, metronidazole or levofloxacin, plus amoxicillin or tetracycline, with or without bismuth, in the same prescription order for 7–14 days); and/or 2) HP infection diagnosis (041.86). The accuracy of disease diagnoses in the NHI database has been studied previously. Agreements between claim data and medical records are moderate to substantial, with Kappa values ranged from 0.55 to 0.86 [37].

Baseline characteristics between never users and ever users were compared by Student's t test for age and by Chi-square test for other variables.

The incidence density of esophageal cancer was calculated for never users and ever users and for the tertiles of cumulative duration of metformin therapy. The numerator was the case number of incident esophageal cancer during follow-up, and the denominator was the person-years of follow-up. Follow-up started on the first day of the use of antidiabetic drugs and ended on December 31, 2011, at the time of a new diagnosis of esophageal cancer, or on the date of death or the last reimbursement record.

The baseline characteristics shown in Table 1 were used for creating PS by logistic regression, and the treatment effect was estimated by Cox regression incorporated with IPTW using the PS [23, 24]. Hazard ratios were estimated for ever versus never users and for each tertile of cumulative duration using never users as referent.

Because the baseline characteristics were imbalanced between metformin ever and never users, additional analyses were conducted in a 1:1 PS matched-pair sample (matched sample), which was created by using the Greedy 8 → 1 digit match algorithm as recommended by Parsons [38]. Because the case number of metformin never users was much smaller than the ever users, the matching was based on the case number of never users. This matching method has also been applied in our previous studies [6, 14, 39–41].

If residual systematic differences in baseline characteristics exist, the IPTW approach may not achieve unbiased estimates [42]. A quantitative method based on the calculation of standardized difference has been proposed by Austin and Stuart as a test for balance diagnostics [42]. A value of > 10% might indicate potential confounding from the variable [42]. The standardized differences for all covariates were calculated for both the original sample and the matched sample [42].

Traditional Cox regression models were created to evaluate the separate effect of infection of HP, EBV, HBV and HCV, and the joint effect of metformin and HP infection by categorizing the patients into 4 subgroups: 1) metformin (−)/HP infection (+), treated as the referent group; 2) metformin (+)/HP infection (+); 3) metformin (−)/HP infection (−); and 4) metformin (+)/HP infection (−). These models were created by setting an entry date on January 1, 2006, and followed patients without esophageal cancer before this date for 6 years until December 31, 2011.

Analyses were conducted using SAS statistical software, version 9.3 (SAS Institute, Cary, NC). *P* < 0.05 was considered statistically significant.
